# Phosphodiesterase‐5 Inhibitor Use Masking a Necrotizing Syphilitic Infection: A Rare Differential Diagnosis of Priapism

**DOI:** 10.1155/criu/6685174

**Published:** 2026-09-17

**Authors:** Dimitrios M. Papadimopoulos, Apostolos Chasapis, Konstantinos Sitaridis, Evangelos Arapis, Nikolaos Kalogeras, Apostolos Papalakis

**Affiliations:** ^1^ Department of Urology, General Hospital of Thessaloniki “O Agios Dimitrios”, Thessaloniki, Greece

## Abstract

**Background:**

Acute penile enlargement is often presumed to indicate priapism, particularly after Phosphodiesterase‐5 inhibitor use. However, alternative diagnoses, including infectious and necrotizing processes, should be considered—especially in patients with a history of unprotected sex—to avoid delayed or inappropriate management.

**Case Presentation:**

A 43‐year‐old male with a history of intravenous drug use presented with acute penile swelling and leukocytosis (20,500/*μ*L) following unprotected sexual intercourse and tadalafil consumption. Although priapism was suspected, corporal blood gas analysis demonstrated oxygenation and pH levels that excluded ischemic priapism. The patient was admitted to the clinic, and empiric intravenous antibiotics (piperacillin/tazobactam and clindamycin) were initiated. Despite laboratory improvement, penile edema and erythema progressed with early necrotic changes. Postoperative history obtained from a relative revealed prior inadequately treated syphilis, subsequently confirmed by serologic testing. *Τ*he patient underwent surgical debridement of necrotic tissue surrounding the corporal bodies. He was discharged and referred to a specialized center for syphilis treatment. The patient recovered without further complications and demonstrated preserved erectile function at 1‐month follow‐up.

**Conclusion:**

Acute penile enlargement following Phosphodiesterase‐5 inhibitor use is not necessarily attributable to priapism and may instead be caused by infectious or necrotizing etiologies. Prompt recognition, initiation of antimicrobial therapy, and timely surgical intervention are critical to preserve penile tissue and erectile function.

## 1. Introduction

Acute penile enlargement is a relatively common urological presentation with a broad differential diagnosis, including priapism, infection, filariasis, penile fracture, paraphimosis, trauma, adverse drug reactions, and dermatological conditions such as contact dermatitis [1, 2]. Among these conditions, priapism represents a urological emergency requiring prompt recognition and treatment, as delayed management may result in smooth muscle necrosis, corporal fibrosis, and permanent erectile dysfunction [3, 4]. Similarly, paraphimosis constitutes a true urological emergency, as prolonged constriction of the glans may compromise vascular perfusion and progress to ischemia, necrosis, and, in severe cases, gangrene [5]. Nevertheless, acute penile enlargement does not invariably indicate priapism, and alternative etiologies should be considered to ensure accurate diagnosis and appropriate management.

Infectious and necrotizing conditions of the penis are rare but important differential diagnoses in patients presenting with acute penile edema. Syphilis is not commonly considered among the initial differential diagnoses in such presentations. However, its incidence has increased in recent years, with a 28.6% increase reported between 2020 and 2021 [6]. Moreover, syphilis infection rates continue to rise worldwide, with 176,713 cases reported in 2021 [6].

Syphilis, often referred to as the “great imitator” of dermatological diseases, may present with a wide range of atypical clinical manifestations, a phenomenon attributed to the pathomorphosis of the disease [7]. The pathomorphosis of syphilis may be natural or iatrogenic and has been associated with several factors, including antibiotic or other chemotherapeutic treatment for concomitant diseases; chronic alcohol consumption; alterations in individual immune responsiveness related to intoxication or infection, including AIDS; and advanced age [7]. Among its atypical manifestations, soft tissue necrosis may mimic priapism or other acute urological emergencies, such as Fournier′s gangrene. Failure to recognize these atypical presentations may result in inappropriate interventions and subsequent tissue loss.

We report a case of necrotizing syphilitic infection presenting as acute penile enlargement following Phosphodiesterase‐5 (PDE‐5) inhibitor use. This case showcases that the association between the use of PDE‐5 inhibitors and ischemic priapism can act as a diagnostic pitfall in patients presenting with marked penile edema. Therefore, it highlights the importance of careful diagnostic evaluation, including corporal blood gas analysis, and illustrates the role of early antimicrobial therapy and surgical intervention in preserving tissue and function.

## 2. Case Presentation

A 43‐year‐old male with a history of intravenous drug use presented to the emergency department with acute penile swelling after unprotected sexual intercourse and tadalafil consumption the day prior. He was afebrile on admission, without any systemic symptoms. Laboratory evaluation revealed leukocytosis (20,500/*μ*L; reference range 4000–10,000/*μ*L). The patient denied any prior hematologic disorders, penile trauma, or intracavernosal injections.

On physical examination, the penis was diffusely swollen, enlarged, and erythematous. Corporal blood gas sampling was attempted to assess for ischemic priapism. However, the procedure was technically challenging because of the marked penile edema. The obtained sample demonstrated findings that were not suggestive of either ischemic or nonischemic priapism (pO_2_: 42 mmHg; pCO_2_: 48 mmHg; and pH: 7.38). Given these findings, which suggested an etiology other than priapism, further penile imaging, including assessment of cavernosal arterial flow with Doppler ultrasonography, was not performed. The patient was subsequently admitted to the urology department, and empiric intravenous antibiotic therapy with piperacillin/tazobactam and clindamycin was initiated at standard therapeutic doses. Several hours later, the patient developed a high‐grade fever (38.8°C), which was managed with paracetamol and parecoxib.

**Figure 1:**
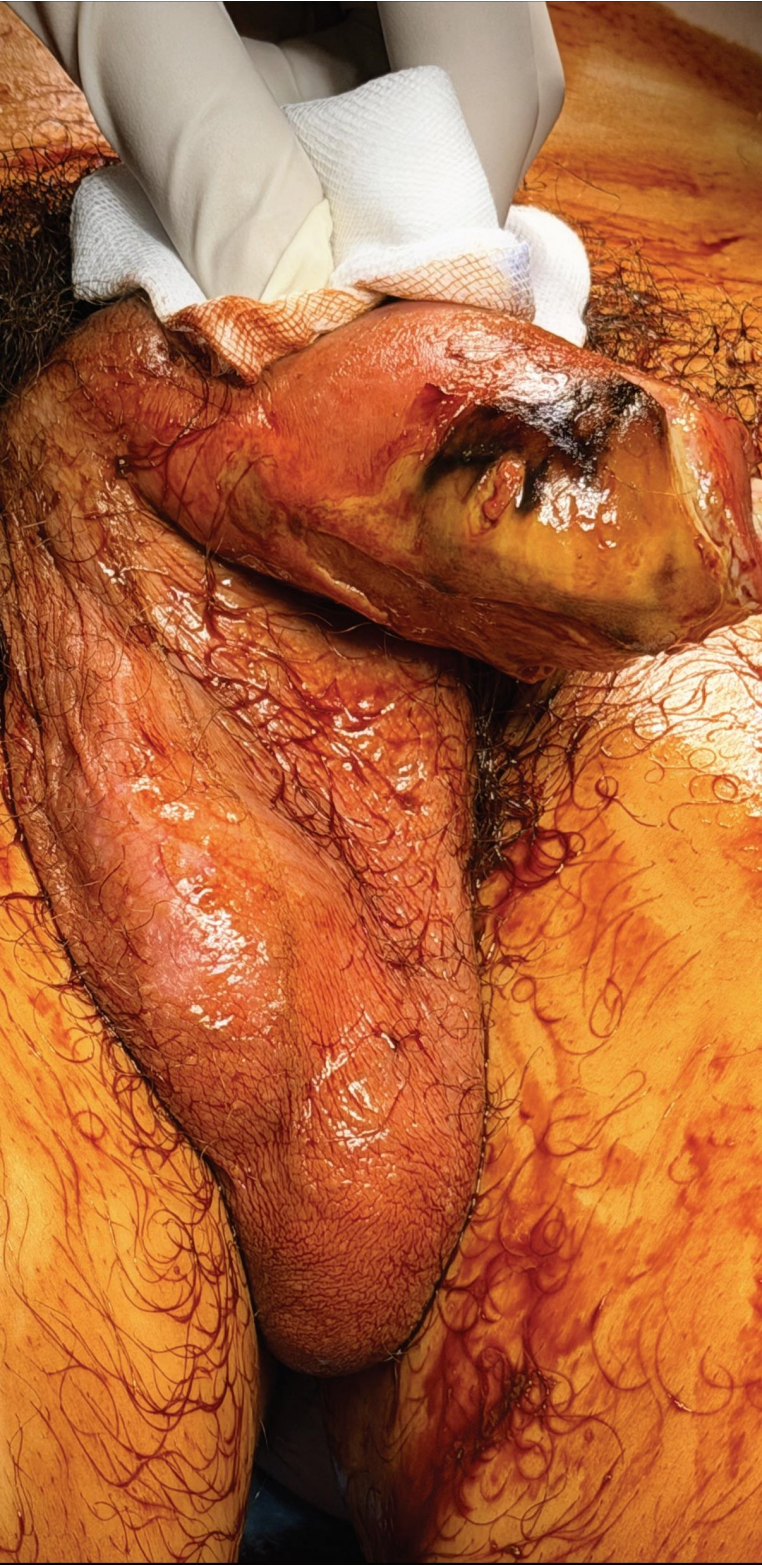
Intraoperative picture of the necrotizing tissue on the right side of the penile shaft and the prepuce.

**Figure 2:**
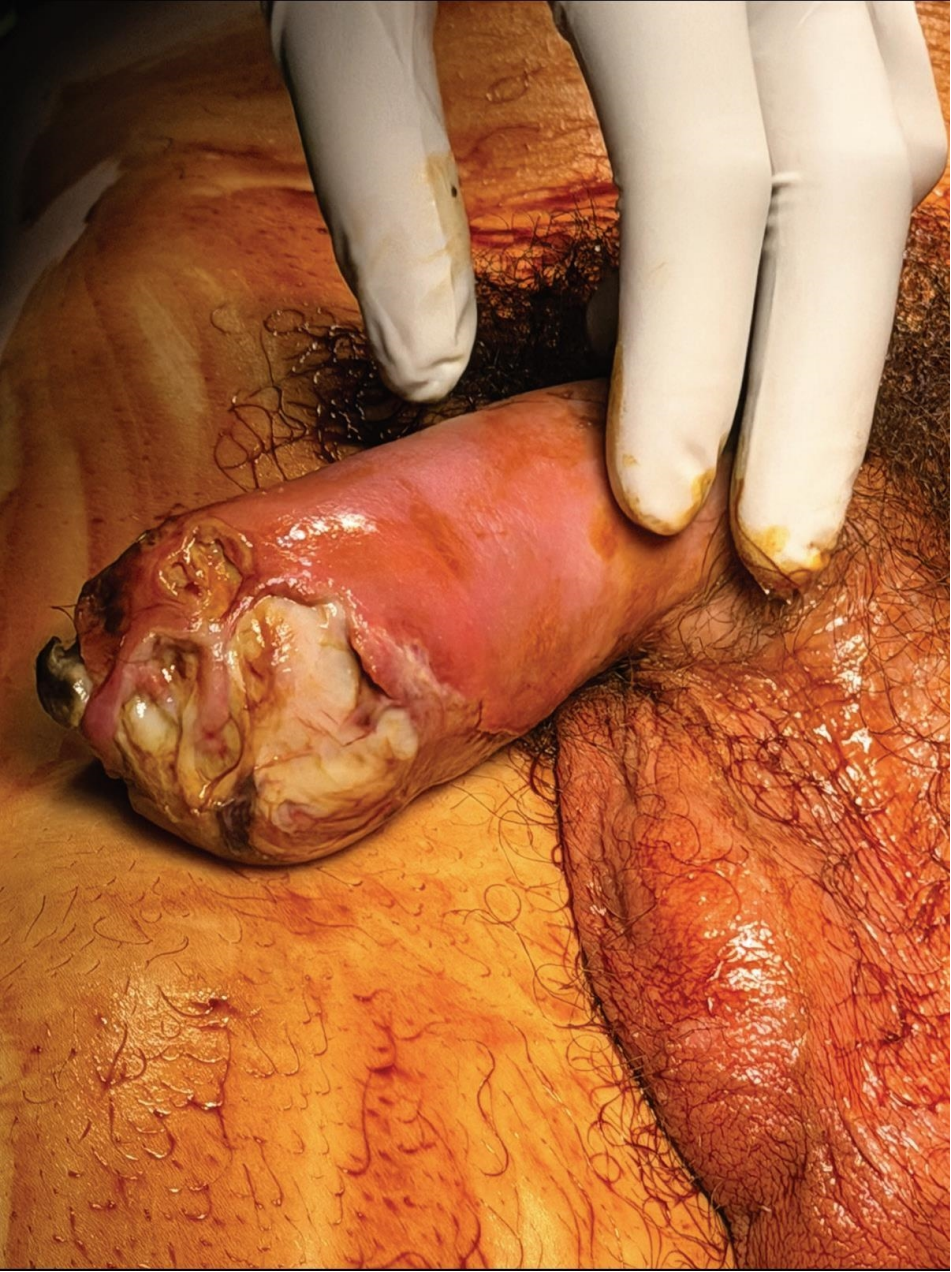
Intraoperative picture of the necrotizing tissue on the left side of the penile shaft and the prepuce.

Despite improvement in the patient′s fever and leukocyte count, penile edema and erythema progressively worsened over the following 2 days, with the development of early necrotic changes along the penile shaft (Figures 1 and 2). Urgent surgical exploration and debridement of the necrotic tissue were, therefore, performed. Intraoperatively, necrosis was confined to the penile skin and subcutaneous tissues, with preservation of the corporal bodies and corpus spongiosum. The Dartos layer and Buck′s fascia were also unaffected. All nonviable tissue was excised, and the viable preputial margins were circumferentially reapproximated without tension, without the need for additional reconstructive procedures, resulting in complete exposure of the glans.

Intraoperative tissue cultures were obtained for microbiological analysis, including Gram‐positive, Gram‐negative, fungal, and anaerobic cultures. Cultures were positive for *Staphylococcus aureus*, with a bacterial load of 10^6^ CFU/mL. Following removal of the prepuce, a previously concealed ulceration of the glans was identified, which had been obscured preoperatively by the surrounding inflamed tissue.

Postoperatively, additional history obtained from a relative visiting the patient for the first time reported that the patient had a prior diagnosis of syphilis that had not been fully treated, which had not been disclosed at admission. Therefore, a serological VDRL test was ordered, and the results showed a titer of 1:64, indicating active syphilis infection and providing an explanation for the necrotizing changes. Subsequent immunohistochemistry (IHC) analysis on surgical biopsies/cultures came back positive for *Treponema pallidum*, confirming the presence of the spirochete within the lesion. The patient was also tested for HIV (HIV‐1/2 antibodies), which were negative.

Over the following days, the patient remained afebrile and laboratory values normalized, with the leukocyte count decreasing from 20,500 to 7600/*μ*L and C‐reactive protein from 44 to 1 mg/dL (reference range < 0.5 mg/dL). He was discharged on Postoperative Day 4 and referred to a specialized center for comprehensive syphilis management. The patient received weekly benzathine Penicillin G 2.4 million units IM for 3 weeks, with good clinical response.

**Figure 3:**
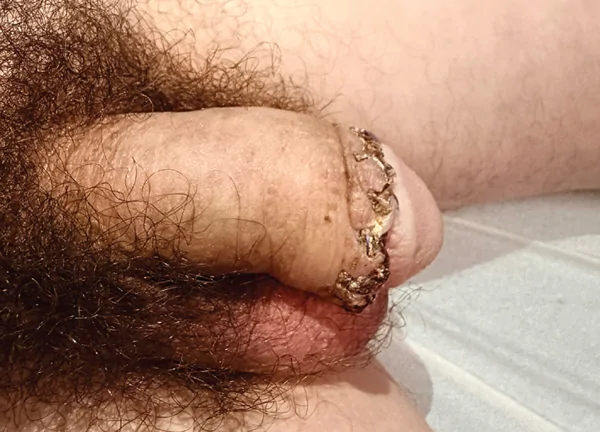
Postoperative Day 30. Reduction of edema and inflammation. No necrotizing tissue remaining.

**Figure 4:**
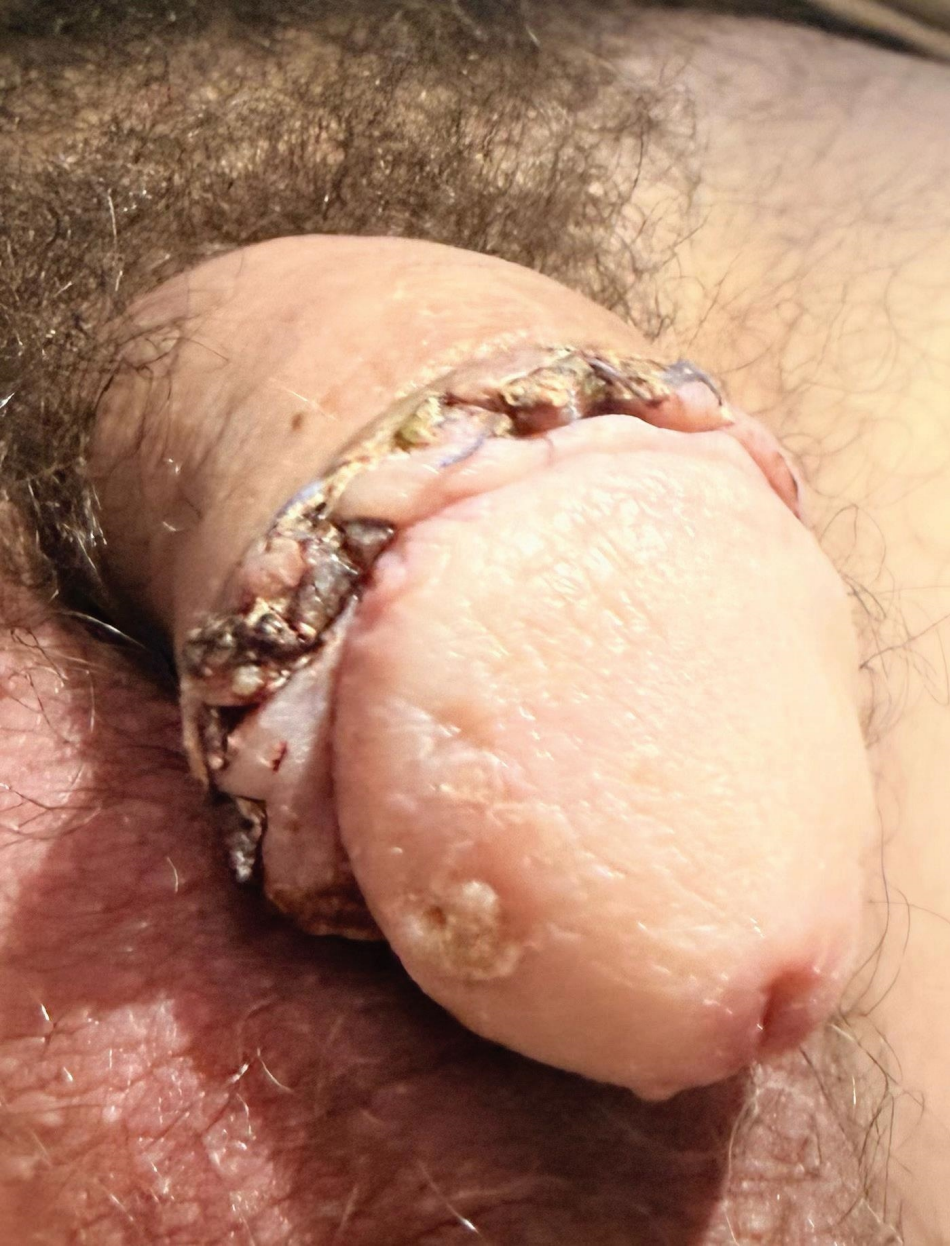
Postoperative Day 30. Anterior view. Visible healing ulcer on the glans penis.

At the 1‐month follow‐up, the inflammatory changes had completely resolved, whereas the glans ulcer had significantly contracted, with ongoing epithelialization and no evidence of residual necrosis. The patient reported the return of spontaneous erections (Figures 3 and 4). To objectively assess erectile function, the International Index of Erectile Function‐5 (IIEF‐5) questionnaire was administered at discharge, retrospectively assessing erectile function prior to the acute infection, and repeated at the 1‐month follow‐up. Notably, there was no change in the total IIEF‐5 score before and after the infection, with a score of 23/25 recorded at both assessments, confirming preservation of erectile function.

## 3. Discussion

Acute penile enlargement is frequently associated with priapism, particularly in the context of PDE‐5 inhibitor use. Although PDE‐5 inhibitors have an overall favorable safety profile, their direct causal role in the development of ischemic priapism has increasingly been questioned in contemporary urological literature, as most reported cases occur in the presence of underlying predisposing factors, such as sickle cell disease, other hemoglobinopathies, concomitant anticoagulant use, or recreational drug use, rather than following drug exposure alone. Nevertheless, the temporal association between PDE‐5 inhibitor use and acute penile swelling may represent a diagnostic pitfall. Penile enlargement may instead reflect an underlying infectious or necrotizing process, emphasizing the importance of maintaining a broad differential diagnosis, particularly in the presence of local inflammatory changes or systemic signs of infection.

In the context of acute urological emergencies, the most critical entity to consider is Fournier′s gangrene. As reviewed by Chernyadyev et al. [8], Fournier′s gangrene represents a hyperacute, polymicrobial necrotizing fasciitis of the perineal and genital regions that demands immediate fluid resuscitation, broad‐spectrum empiric antibiotics, and emergent surgical debridement. Although true Fournier′s gangrene carries a catastrophic mortality rate if left untreated, distinguishing it from localized, atypical infectious processes—such as a phagedenic syphilitic ulcer—is vital for guiding long‐term antimicrobial stewardship and avoiding overly aggressive, mutilating surgery when localized debridement suffices.

Sexually transmitted infections (STIs), particularly syphilis, remain an important global public health concern. The incidence of syphilis has increased significantly in recent years, affecting populations across all socioeconomic levels. Syphilis is well known for its ability to mimic a wide range of dermatologic conditions. For example, Singh et al. [9] reported a case of secondary syphilis presenting with penile edema and lichenoid plaques on the scrotum, whereas penile edema has also been described as a manifestation of syphilitic or Follmann′s balanitis in the primary stage, usually resolving with the chancre. Sardinha et al. [10] analyzed 25 patients with cord‐like lesions on the penis, confirming syphilis in 19 cases.

These reports, along with the present case, illustrate the fact that many physicians may not consider syphilis unless it presents with its classical three‐stage clinical pattern. Modern literature emphasizes that *T. pallidum* routinely masquerades as distinct noninfectious pathologies, severely challenging routine diagnostic algorithms. For instance, syphilis can present as a striking oncological mimic. Shimoda et al. [11] documented a case of secondary syphilis closely mimicking penile cancer, complete with local induration, regional lymphadenopathy, and pseudometastases in the lungs. Furthermore, the atypical presentations of the disease extend beyond solid tumors; Maci et al. [12] illustrated a case where malignant syphilis mimicked lymphoma, presenting with systemic B symptoms and extensive lymphadenopathy that complicated radiological and flow cytometry analyses. However, if left untreated, syphilis can lead to severe complications, including blindness, paralysis, and death. Physicians should, therefore, maintain a high index of suspicion for syphilis in patients presenting with atypical penile lesions, particularly in those with a history of unprotected sexual intercourse. This is especially relevant for cases of acute penile edema, which are often misattributed to priapism.

Regarding the staging of the disease, highly destructive or necrotizing penile lesions can pose a significant diagnostic challenge, mimicking malignant secondary syphilis (lues maligna) or tertiary gummatous syphilis. However, lues maligna typically presents with widespread, multiple ulceronecrotic lesions and prominent systemic manifestations, which were completely absent in our patient at admission. Furthermore, the literature demonstrates a fundamental epidemiological link between severe, necrotizing, or malignant variants of syphilis and underlying immunosuppression, frequently serving as the initial presentation of an undiagnosed HIV infection, as thoroughly reviewed by Maci et al. [12] and documented by Devkota et al. [13]. In such coinfected patients, a compromised cellular immune response accelerates *T. pallidum* dissemination and exacerbates local tissue infarction. Similarly, tertiary gummas exhibit a chronic, indolent course rather than an acute urological emergency.

Therefore, based on the localized presentation, the intraoperative identification of a single glans ulcer, and the rapid progression following secondary bacterial invasion, this case is best characterized as a primary phagedenic chancre. Crucially, our patient tested negative for HIV, demonstrating that extensive, tissue‐destructive treponemal necrosis can still manifest in immunocompetent individuals. The severity of tissue destruction was likely driven by anaerobic superinfection, potentially exacerbated by a tissue‐destructive immune response related to the patient′s history of incomplete prior treatment or reinfection.

In this patient, the initial evaluation revealed penile edema and erythema, accompanied by leukocytosis and elevated inflammatory markers. Although real‐world data indicate that priapism following oral PDE‐5 inhibitor use is rare [14], the recent use of a PDE‐5 inhibitor in conjunction with penile edema initially raised suspicion for priapism. However, corporal blood gas analysis was not strongly suggestive of ischemic priapism, indicating the possibility of an alternative etiology. It should be noted that corporal blood aspiration in such cases may be technically challenging because marked tissue edema and inflammation can compress the corpora cavernosa and hinder adequate sampling.

In the present case, penile Doppler ultrasonography was not performed following the blood gas analysis, as priapism was considered unlikely and alternative causes of penile edema were suspected. Nevertheless, penile Doppler ultrasonography can provide objective and clinically relevant information regarding the preservation of cavernosal arterial flow. Therefore, the omission of this imaging modality represents a limitation of the diagnostic pathway in this case.

This case highlights two important clinical considerations. First, corporal blood gas analysis may assist in differentiating priapism from penile infectious processes, even in the presence of marked penile swelling. Second, clinicians should be aware that substantial edema and tissue distortion may render corporal aspiration technically difficult, and blood gas results should, therefore, be interpreted in conjunction with the overall clinical presentation rather than in isolation.

Despite initiation of broad‐spectrum antimicrobial therapy, penile swelling and erythema progressed, necessitating surgical exploration. Early debridement prevented extension to the corporal bodies and contributed to the preservation of erectile function. As emphasized in the framework of necrotizing genital infections by Chernyadyev et al. [8], prompt surgical exploration and tailored antimicrobial therapy remain the cornerstones of treatment to prevent rapid, fascial‐plane disease propagation. Necrotizing penile infections, although uncommon in developed countries, are more frequently observed among individuals with factors such as intravenous drug use, diabetes mellitus, immunosuppression, or poor hygiene.

Syphilis continues to demonstrate diverse and occasionally atypical clinical manifestations. Although primary disease typically presents with a painless chancre, delayed recognition or incomplete treatment may result in unusual presentations. In this patient, the concealed glans ulcer represented the primary infectious focus, whereas the surrounding inflammatory and necrotizing changes obscured the cause.

The patient′s postoperative course was favorable, with normalization of inflammatory markers and complete resolution of penile inflammation and glans ulceration at the 1‐month follow‐up. Early recognition and timely surgical intervention were crucial in preserving penile anatomy and function. Following definitive confirmation of *T. pallidum* infection by intraoperative IHC, the patient successfully completed guideline‐compliant therapy consisting of three weekly intramuscular doses of benzathine penicillin G (2.4 million units), appropriate for syphilis of unknown duration or late latent syphilis.

It should be acknowledged that the use of the IIEF‐5 questionnaire in this case did not fully align with its validated application in routine clinical practice, as the baseline assessment was performed retrospectively and repeated after a relatively short follow‐up period of 1 month. Nevertheless, given the unusual clinical presentation and the absence of pre‐existing erectile dysfunction symptoms prior to hospitalization, the authors considered this approach the most feasible method for obtaining an estimate of baseline erectile function and patient satisfaction, allowing subsequent assessment following treatment.

This case highlights several important clinical considerations. Necrotizing infections, including syphilis, should be considered in patients presenting with progressive penile swelling, particularly in the presence of elevated inflammatory markers, systemic symptoms, or progression of local lesions despite initial therapy. A history of PDE‐5 inhibitor use in conjunction with unprotected sexual intercourse, particularly among men who have sex with men, may further raise clinical suspicion for syphilis or other STIs. These factors may be interconnected through a well‐documented behavioral association between PDE‐5 inhibitor use [14] and engagement in high‐risk sexual practices. This association has been consistently reported in the literature, including a large review, cross‐sectional studies, and community‐based survey data [15–17].

Additionally, it underscores the inherent unreliability of patient‐reported histories, particularly concerning STIs and substance use. Due to social stigma, embarrassment, or fear of judgment, patients frequently underreport or completely conceal high‐risk behaviors and past medical diagnoses upon admission. In this instance, the crucial information regarding a prior, incomplete syphilis treatment was only uncovered postoperatively through a collateral history provided by a relative.

As the academic community continues to encourage the publication of educational case reports, this case serves as a scientifically rigorous reminder that clinicians must look past the initial clinical narrative, maintain a high index of suspicion, and rely on objective pathological tools to guide successful management.

## 4. Conclusion

Necrotizing syphilitic infection can mimic priapism, even in the context of PDE‐5 inhibitor use. Corporal blood gas findings not indicative of priapism should prompt consideration of alternative diagnoses. Necrotizing infections, including syphilitic infection, should be considered in patients with progressive penile swelling, particularly when laboratory markers are elevated, systemic symptoms are present, or when lesions advance despite initial therapy. Thorough history‐taking and appropriate serological testing are essential to identify unusual infectious etiologies and guide timely intervention. Early recognition, antimicrobial therapy, and timely surgical intervention are critical to preserve tissue and function.

## Funding

No funding was received for this manuscript.

## Consent

Written informed consent was obtained from the patient for publication of this case report and any accompanying images. The patient has been assured that all personal identifiers have been removed and that confidentiality will be maintained.

## Conflicts of Interest

The authors declare no conflicts of interest.

## Data Availability

The data that support the findings of this study are available from the corresponding author upon reasonable request.
